# *Astragalus* polysaccharide modulates the gut microbiota and metabolites of patients with major depressive disorder in an *in vitro* fermentation model: a pilot study

**DOI:** 10.3389/fnut.2025.1587742

**Published:** 2025-04-28

**Authors:** Yuwei Mi, Chen Lin, Huowang Zheng, Ying Wu, Yanbin Hou, Jieqiong Hu, Jiaxin Mao, Ni Dai, Xiaoqiong Li, Zhongze Lou, Yunxin Ji

**Affiliations:** ^1^Department of Psychosomatic Medicine, The First Affiliated Hospital of Ningbo University, Ningbo, Zhejiang, China; ^2^State Key Laboratory for Managing Biotic and Chemical Threats to the Quality and Safety of Agro-products, Zhejiang Academy of Agricultural Sciences, Hangzhou, China; ^3^School of Medicine, Ningbo University, Ningbo, Zhejiang, China

**Keywords:** *Astragalus* polysaccharide, *Lactobacillus plantarum* PS128, depression, gut microbiota, tryptophan, metabolites, *in vitro* fermentation

## Abstract

Previous studies have found that *Astragalus* Polysaccharide (APS) and *Lactobacillus plantarum* PS128 (PS128) have potential antidepressant effects, but their effects on the gut microbiota and metabolites of major depressive disorder (MDD) are still unclear. We examined the effect of APS on gut microbiota and metabolites of first-episode and drug naïve MDD patients using *in vitro* fermentation, and further explored whether PS128 could enhance the utilization ability of APS. Fresh fecal samples from 15 MDD patients were collected, and analyzed for differences in gas production, gut microbiota, and tryptophan (Trp) related metabolites after 48 h of fermentation. APS fermentation increased the abundance of *Bifidobacterium* and decreased the abundance of *Lachnoclostridium* (*p* < 0.05). APS also increased total gas production and levels of indole lactic acid (ILA), Trp, and 5-hydroxytryptophan (5-HTP) (*p* < 0.05). Compared with APS, APS with PS128 synbiotics fermentation increased the abundance of *Lactobacillus* (*p* < 0.05), reduced total gas production and percentages of CO_2_, H_2_, and H_2_S (*p* < 0.05), and to some extent increased the levels of ILA, Trp, and 5-HTP, although not statistically significant (*p* > 0.05). Correlation analysis showed *Bifidobacterium* was positively correlated with ILA, Trp and 5-HTP; On the contrary, *Lachnoclostridium* was negatively correlated with ILA, Trp, and 5-HTP. All these results suggest that APS could regulate gut microbiota structure and Trp related metabolites in MDD patients; Compared to APS, APS and PS128 synbiotic fermentation could reduce gas production but shows limited ability to modulate gut microbiota structure or Trp related metabolites in MDD patients.

## 1 Introduction

Major depressive disorder (MDD) is one of the most common chronic and distressing diseases and there are more than 350 million people worldwide who struggle with MDD every day (1). Currently, the commonly used clinical antidepressant drugs, such as selective serotonin reuptake inhibitors (SSRIs), are associated with low response rates, gastrointestinal dysfunction, sexual dysfunction, and other issues (2). Therefore, there is an urgent need to explore an effective and low-side-effect treatment method for MDD.

The gut microbiota plays a vital role in bidirectional signaling between the gut and the central nervous system, known as microbiota-gut-brain axis (MGBA) (3). Recently, accumulating evidence indicates gut microbiota dysbiosis is involved in pathogenesis of MDD (4–6). For example, germ-free mice exhibited depressive-like behavior underwent fecal microbiota transplantation from MDD patients (7). Supplemental *Bifidobacterium breve* Bre1025 reversed stress-induced depressive behaviors in mice and restored indole lactic acid (ILA) levels in the gut and brain (8). Gut microbiota dysbiosis could affect the neurobiology of MDD through host Tryptophan (Trp) metabolism mediated pathways (9). Thus, modulating the dysbiosis of gut microbiota is expected to become a new approach for MDD.

One approach to modulate the microbiota is to use prebiotics. Prebiotics are non-digestible compounds that selectively stimulate the growth or activity of beneficial bacteria in the gut to improving the health of the host with few side effects (10). *Astragalus* Radix (AR, Huangqi in Chinese) has the effect of resolving depression (11) and its related formulations are widely used in traditional Chinese medicine to treat MDD (12). Animal studies have shown that AR can reverse depressive-like behavior in depressed mice, demonstrating potential antidepressant effects (13, 14). *Astragalus* Polysaccharide (APS), one of the main active substance of AR, is a potential prebiotic that has been proven to enhance immune (15), anti-neuroinflammatory (16) and anti-oxidant functions (17). An *in vitro* study on the feces of patients with type 2 diabetes found that APS fermentation could increase the abundance of *Lactobacillus* and *Bifidobacterium*, and increase the levels of propionic acid, in all-trans-retinoic acid and thiamine, which indicated APS may alleviate type 2 diabetes by intestinal gut microbes and metabolites (18). However, the impact of APS on the intestinal microbiota and intestinal metabolic profile in MDD patients is still unclear.

Another safe and applicable approach to modulate the microbiota is to use probiotics (19). Psychobiotics are living microorganisms that can provide health benefits for patients with mental illness when ingested in adequate amounts (20). *Lactobacillus plantarum* PS128 (PS128) is a psychobiotic isolated from spontaneously fermented mustard greens in Taiwan. Animal experiments indicated that PS128 alleviated anxiety-and depressive-behaviors in stress-induced mice and regulated dopamine and serotonin levels in the brain (21, 22). Clinical studies further indicated that PS128 supplementation not only improved depressive symptoms in patients with MDD (23), but also improved depressive symptoms and sleep quality in insomnia patients (24). These studies suggest that the administration of PS128 has a great potential for treating patients with MDD.

Prebiotics are fermentation substrates of probiotics, which are used together with probiotics to improve host health (25). The synbiotic combination of prebiotics and probiotics is considered to have greater benefits for hosts than either prebiotics or probiotics alone (26). An *in vitro* study found that the consumption of probiotics could effectively enhance the utilization of grape seed extract, increase the level of short-chain fatty acids (SCFA), and restore the dysbiosis of gut microbiota (27). Nevertheless, the utilization of probiotics may be affected by the complexity of prebiotic components and structures (28). A recent *in vitro* work found that *Lacticaseibacillus paracasei* K56 had better ability to utilize galactooligosaccharide than polydextrose and xylooligosaccharide in obesity patients (25). Hence, it is worth to explore the optimal combination of prebiotics and probiotics to improve mental health of MDD patients.

*In vitro* fermentation is widely used and has the advantages of low cost, being fast, and high efficiency. *In vitro* fermentation model is recommended to preliminary research on probiotics and prebiotics to predict their *in vivo* function (25). To our knowledge, data on *in vitro* fermentation of prebiotics and/or probiotics for patients with MDD is lacking. In addition, there are no clinical studies on the *in vivo* application of APS with PS128 synbiotics (APS**_**PS128) in MDD patients. To this end, one of our study objectives is to use an *in vitro* simulated fermentation model to elucidate the moderation effect of APS on gut microbiota and metabolic profile of MDD patients by combining 16S sequencing technology and metabolomics. Another study objective is to elucidate whether the consumption of PS128 could enhance the utilization ability of APS in MDD patients.

## 2 Materials and methods

### 2.1 Study design and feces collection

This cross-sectional pilot study was conducted between July 2023 and December 2023, at the First Affiliated Hospital of Ningbo University. All participants met the following inclusion criteria: (1) 18 to 60 years old; (2) Han Chinese; (3) meeting the diagnostic criteria for MDD in the Diagnostic and Statistical Manual of Mental Disorders (Fifth Edition, DSM-5); (4) first acute episode without taking any psychotropic medication before, such as antidepressants, anxiolytics, or antipsychotics; (5) the 17-item of Hamilton Depression Scale (HAMD) score ≥ 17; and (6) No probiotics or antibiotics were consumed in 1-month before enrollment in the study. Exclusion criteria: (1) serious physical illness, including central nervous diseases and acute, unstable or life-threatening medical diseases such as cancer and organ failure; (2) alcohol or substance use disorders; (3) pregnant or breastfeeding women; (4) refusal to take part in the study; and (5) consumption of a non-standard diet (e.g., vegan, vegetarian). Finally, a total of 15 patients with first-episode and drug naïve MDD were recruited and their general information and fecal samples were collected. The fecal samples from all 15 patients were subjected to the three different fermentation tests conducted: the CON group, the APS group, and the APS_PS128 group. All included participants provided written informed consent and the study was approved by the Institutional Review Broad of the First Affiliated Hospital of Ningbo University (No. 2022-046A-01).

### 2.2 *In vitro* fermentation test

The *in vitro* fermentation test was conducted as described previously (29). Fresh fecal samples (0.6 g) were added with 6 mL of 0.1 M anaerobic phosphate-bufered saline (pH 7.0). Next, the feces were homogenized to obtain a 10% fecal suspension, which was filtered through sterile gauze to obtain the filtrate. Subsequently, 0.5 mL of the filtrate was inoculated into 5 mL of a sterilized media, which placed in a 37°C incubator and fermented for 48 h. APS was provided by Hengxing Pharmaceutical Research Institute (Hefei, China). IdentifiPS128 probiotic product was purchased from Asian Probiotics and Prebiotics Corporation (Shanghai, China). Probiotic product powder was used for enumeration in De Man-Rogosa Sharpe broth (MRS) medium and colony isolation on MRS agar plate at 37°C for 18 and 48 h, respectively. The isolated single colony was enumerated and identified as PS128 by 16S rDNA sequencing according to the procedures provided in a previous report (30). The composition of the yeast extract–casein hydrolysate–fatty acid medium (YCFA) modifed growth medium used in three fermentation groups is listed in Table 1. The medium was sterilized at 121°C for 30 min. The three fermentation groups were as follows: the CON group (YCFA), the APS group (YCFA + 8 g/L APS), and the APS_PS128 group (YCFA + 8 g/L APS + 10^9^ CFU/mL PS128).

**Table 1:** YCFA medium formula.

YCFA medium formula	g/L
Tryptone	10
Yeast extract	2.5
L-cysteine	1.0
NaCl	0.9
CaCl_2_·6H_2_O	0.009
KH_2_PO_4_	0.45
K_2_HPO_4_	0.45
MgSO_4_·7H_2_O	0.09
Hemoglobin	5.0
Resazurin	1.0
Vitamin I (uL)	200

Vitamin I: VH 0.05 mg, VB12 0.05 mg, p-aminobenzoic acid 0.15 mg, folic acid 0.25 mg, and pyridoxamine 0.75 mg.

### 2.3 Determination of gas measurement

Following a 48 h in intro fermentation period, total gas production volume and percentage of CO_2_, CH_4_, H_2_, and H_2_S were evaluated using a gas analyzer (HL-QT01, Beiduokang High-tech Co. Ltd., Hangzhou, China). Firstly, the gases from the control medium (without inoculum) were tested to calibrate the analyzer. Then, the gases in each processing medium were released into the analyzer, and a gas sensor measured the gas production volume. The results were analyzed using the “MultiGas Analyzer. exe” software.

### 2.4 Gut microbiome profiling by 16S rRNA sequencing

After a 48 h fermentation period, 1.6 mL of the sample was centrifuged for 10 min (8,000 g, 4°C), the supernatant was removed, and the precipitated sample was sent to Major Biotechnology in Shanghai, China, for 16S rRNA gene sequencing. The total genomic DNA was extracted using the FastPure Stool DNA Isolation Kit (MJYH, Shanghai, China). The quality and concentration of extracted DNA were determined by 1.0% agarose gel electrophoresis and a NanoDrop® ND-2000 spectrophotometer (Thermo Scientific Inc., USA). The primers used in V3-V4 segment amplification were 341F (5’-CCTACGGGAGGCAGCAG-3′) and 806R (5’-GGACTACHVGGGTWTCTAAT-3′). Amplicons were extracted from 2% agarose gel, and purified using the AxyPrep DNA Gel Extraction Kit (Axygen Biosciences, USA), and then quantified using a QuantiFluor™-ST fluorescent quantitative system (Promega, USA). Purified amplicons were pooled in equimolar amounts and paired-end sequenced on an Illumina Nextseq2000 platform (Illumina, San Diego, USA).

After demultiplexing, use fastp (version 0.19.6) to perform quality filtering on the obtained sequence, and merge it with FLASH (version 1.2.7). Then, the high-quality sequences were denoised using DADA2 plugin in the Qiime2 (version 2020.2) pipeline with recommended parameters, which obtains single nucleotide resolution based on error profiles in samples. DADA2 denoised sequences are called amplicon sequence variants (ASVs). To minimize the effects of sequencing depth on alpha and beta diversity measure, the number of sequence from each sample was rarefied to 20,000, which still yielded an average Good’s coverage of 99.09%. Taxonomic assignment of ASVs was performed using the Naive bayes consensus taxonomy classifier implemented in Qiime2 and the SILVA 16S rRNA database (version 138).

The 16S rRNA data are deposited in the NCBI repository, accession number PRJNA1231469. The gut microbiota data were analyzed using the Majorbio platform.^1^ We use Mothur (version 1.30.2) to calculate the alpha diversity index including Ace, Chao1, and Sobs. Due to Ace, Chao1 and Sobs all follow a normal distribution, we perform difference analysis using repeated measures one-way analysis of variance (ANOVA) and Tukey’s *post hoc* test. *β*-Diversity was determined by principal coordinate analysis (PCoA) based on Bray–curtis dissimilarity using QIIME2 and Vegan v2.5.3 package, and statistically examined by Adonis. Linear discriminant analysis (LDA) of effect size (LEfSe) was used to determine the significantly abundant taxa (phylum to genera) of bacteria in different groups (LDA score > 4, *p* < 0.05).

### 2.5 Metabolomic analysis

An ultra-high performance liquid chromatography coupled to tandem mass spectrometry (UHPLC–MS/MS) (ExionLC™ AD UHPLC-QTRAP 6500+, AB SCIEX Corp., Boston, MA, USA) was performed to quantify targeted substances in all samples. A total of 15 tryptophan (Trp) related metabolites were tested. A Waters HSS T3 column (2.1 × 100 mm) and two mobile phases (Phase A, 0.1% formic acid; Phase B, 0.1% formic acid in acetonitrile) were used for chromatographic separation by gradient elution. The column temperature was maintained at 35°C and the injection volume was 1 μL. The mobile phase flow rate was set at 0.30 mL/min and the gradient program was set as follows: 0–1 min (0% B), 1–3 min (0–5% B), 3–5 min (5–10% B), 5–6 min (10–15% B), 6–7 min (15% B), 7–10 min (15–60% B), 10–11 min, (60–100% B), 11–12 min (100% B), 12–12.01 min, (100–0% B), 12.01–15 min, (0% B). The data was collected using positive (negative) multiple reaction mode (MRM) mode. The IonSpray Voltage was 5,500 V (-4500 V) and the source temperature was set at 550°C.

### 2.6 Statistical analyses

Categorical variables were represented as frequencies and percentages and continuous variables were represented as mean±standard error of the mean (M ± SEM). Shapiro–Wilk one-sample test was used to confirm the normality. If continuous variables were normally distributed, differences among three groups were analyzed through repeated measures one-way ANOVA; if variables were not normally distributed, the Friedman test was used. Furthermore, if significant differences were found among three groups, we used Tukey’s *post hoc* test for normally distributed variables and Dunn’s post hoc test for non normally distributed variables to analyze the differences between the two groups. Spearman correlation analysis was used to examine the correlation between the abundance of microbiota and differential metabolites. All statistical analyses were performed using GraphPad Prism 8. Figures and graphs were made with GraphPad Prism 8 and Adobe Illustrator software. All *p*-values were two-tailed with a significance level set at 0.05.

## 3 Results

### 3.1 Clinical information of MDD patients and gas production during *in vitro* fermentation

A total of 15 patients with MDD were recruited in our study, including 4 males (26.7%) and 11 females (73.3%). The average age was 37.5 ± 3.3 years old, BMI was 22.9 ± 1.2 kg/m^2^, and the score of HAMD-17 was 20.3 ± 0.6 (Table 2).

**Table 2:** Basic characteristics of the patients with major depressive disorder.

Subject ID	Age (years)	Gender	Height (m)	Weight (kg)	BMI (kg/m^2^)	HAMD-17
1	18	Male	1.7	57.5	19.90	25
2	41	Female	1.6	75.0	29.30	19
3	32	Female	1.65	47.0	17.26	18
4	31	Female	1.58	78.0	31.24	20
5	37	Male	1.75	66.5	21.71	18
6	45	Female	1.58	56.0	22.43	26
7	49	Female	1.6	55.0	21.48	20
8	60	Female	1.53	55.0	23.50	20
9	27	Female	1.66	58.0	21.05	21
10	27	Female	1.76	95.0	30.67	21
11	34	Female	1.62	51.0	19.43	20
12	54	Female	1.54	64.8	27.32	17
13	33	Female	1.52	41.0	17.75	20
14	54	Male	1.7	67.0	23.18	22
15	21	Male	1.65	45.0	16.53	18
Mean (SEM)	37.5 (3.3)	–	1.6 (0.02)	60.8 (3.6)	22.9 (1.2)	20.3 (0.6)

SEM, standard error of the mean; HAMD-17, 17-item of Hamilton Depression Scale.

**Figure 1:**
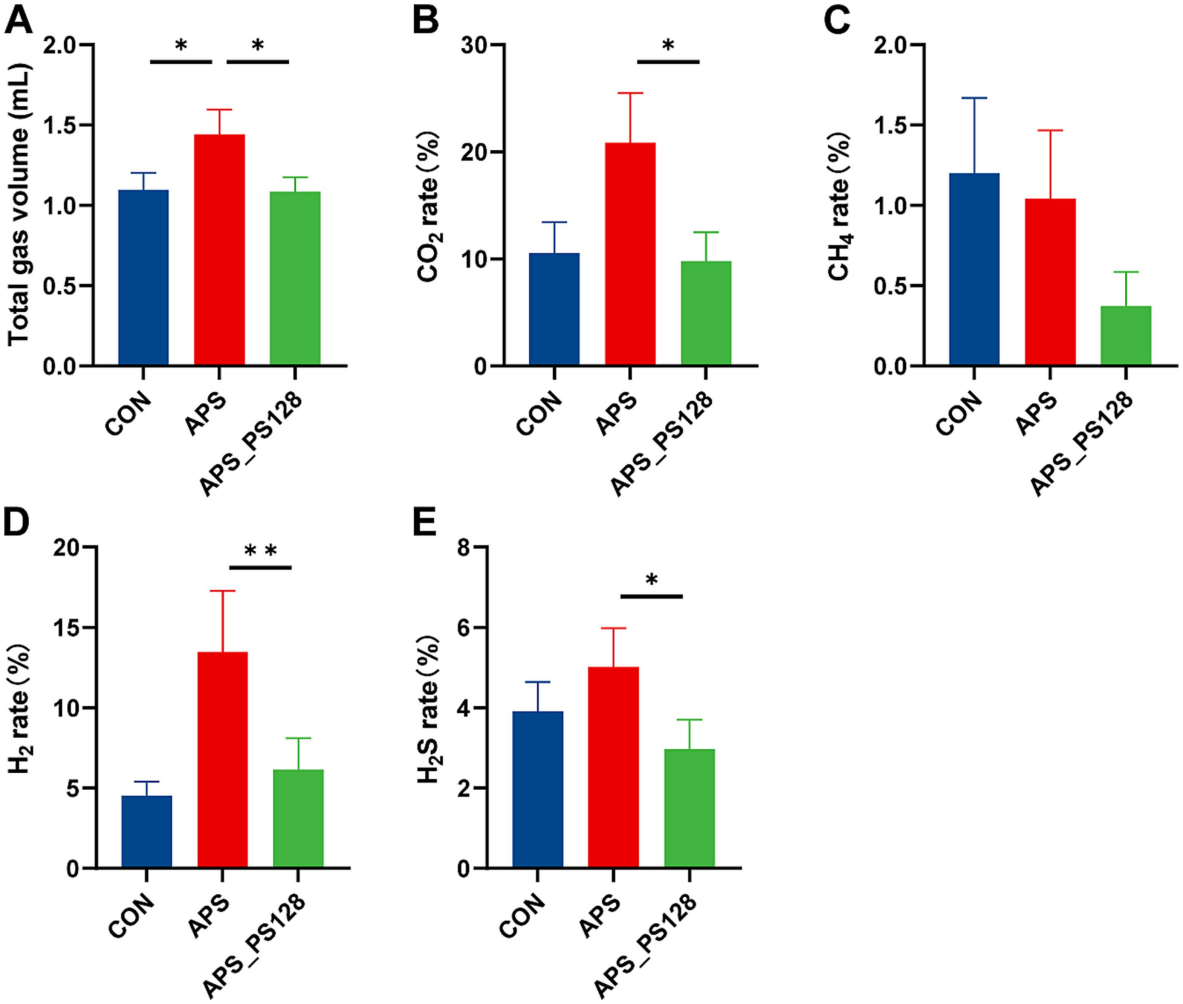
Effects of CON, APS, and APS_PS128 on gas production. **(A)** Total gas production; **(B)** CO_2_ production ratio; **(C)** CH_4_ production ratio; **(D)** H_2_ production ratio; **(E)** H_2_S production ratio. Data are presented as mean ± SEM, and differences are denoted as follows: *0.01 < *p* ≤ 0.05, and **0.001 < *p* ≤ 0.01.

Figure 1 shows gas production in the CON group, the APS group, and the APS_PS128 group. Among the three groups, there was no significant difference in CH_4_ output (*p* > 0.05). The total gas production of the APS group was higher than that of the CON group (*p* < 0.05). Compared with the APS group, the APS_PS128 group had lower total gas production and lower percentages of CO_2_, H_2,_ and H_2_S (all *p* < 0.05).

### 3.2 Impact of APS and APS_PS128 on the gut microbiota

**Figure 2:**
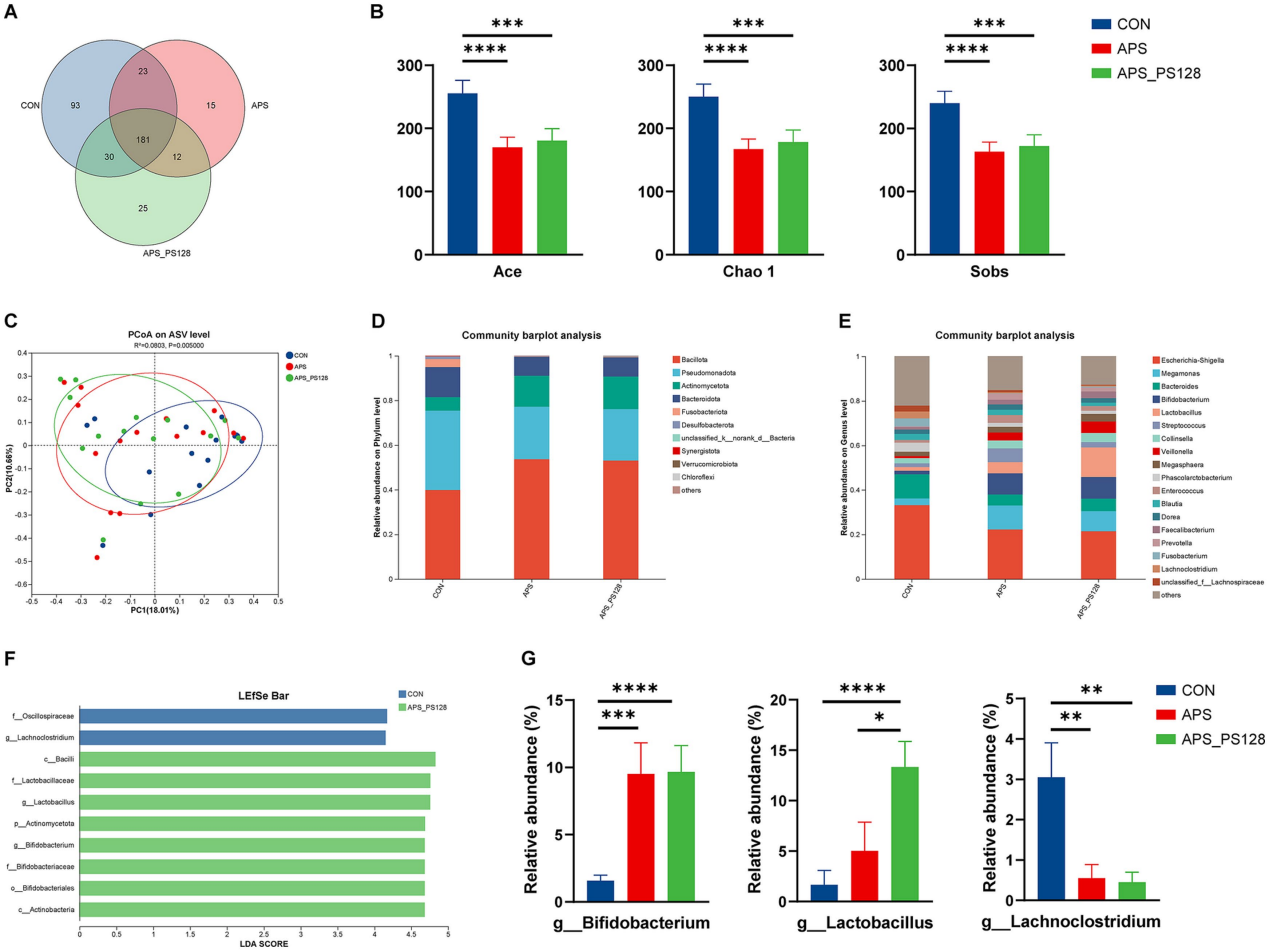
Effects of APS, and APS_PS128 on the gut microbiota in major depressive disorder patients after *in vitro* fermentation. **(A)** Venn diagram at genus level; **(B)** Ace, Chao 1, Sobs indices; **(C)** principal Co-ordinates Analysis (PCoA); **(D)** the bacterial composition at the phylum levels; (D) the bacterial composition at the genus levels; **(F)** LEfSe analyses diagram (at the phylum to the genera levels); linear discriminant analysis (LDA) scores greater than 4 were considered as important differential abundances among groups; **(G)** relative abundance analysis of gut microbiota at the genus levels. Data are presented as mean ± SEM, and differences are denoted as follows: *0.01 < *p* ≤ 0.05, **0.001 < *p* ≤ 0.01, ***0.0001 < *p* ≤ 0.001, and ****p ≤ 0.0001.

According to the Venn diagrams (Figure 2A), the CON group had 327 genera, the APS group had 231 genera, and the APS_PS128 group had 248 genera. Figures 2D,E display the bacterial composition of three groups at the phylum and genus levels, respectively. The richness of the gut microbiota was identified using the Ace, Chao 1, and Sobs indices (Figure 2B). These indices of the APS group (*p* < 0.0001) and the APS_PS128 group (*p* < 0.001) were significantly lower than those of the CON group. There was a significant difference in the gut microbiota structure between the APS group and APS-PS128 group compared to the CON group (Figure 2C) (*p* < 0.05). LEfSe was used to better display microbial communities with significant differences in abundance between groups and an LDA scores greater than 4 was considered as important differential abundances between different groups (*p* < 0.05). *Lactobacillus* and *Bifidobacterium* were the dominant genera in the APS_PS128 group, whereas *Lachnoclostridium* was the dominant genera in the CON group (Figure 2F). As shown in Figure 2G, the relative abundance of *Lactobacillus* in the APS_PS128 group (13.3 ± 2.5%) was significantly higher than that in the CON group (1.7 ± 1.4%, *p* < 0.0001) and the APS group (5.0 ± 2.9%, *p* < 0.05). The relative abundance of *Bifidobacterium* was significantly higher in the APS group (9.5 ± 2.3%, *p* < 0.001) and the APS_PS128 group (9.6 ± 2.0%, *p* < 0.0001) compared with the CON group (9.6 ± 0.4%). In contrast, the relative abundance of *Lachnoclostridium* was significantly lower in the APS group (0.6 ± 0.3%, *p* < 0.01) and the APS_PS128 group (0.4 ± 0.3%, *p* < 0.01) than that in the CON group (3.0 ± 0.9%).

### 3.3 Effects of APS and APS_PS128 on Trp related metabolites

**Figure 3:**
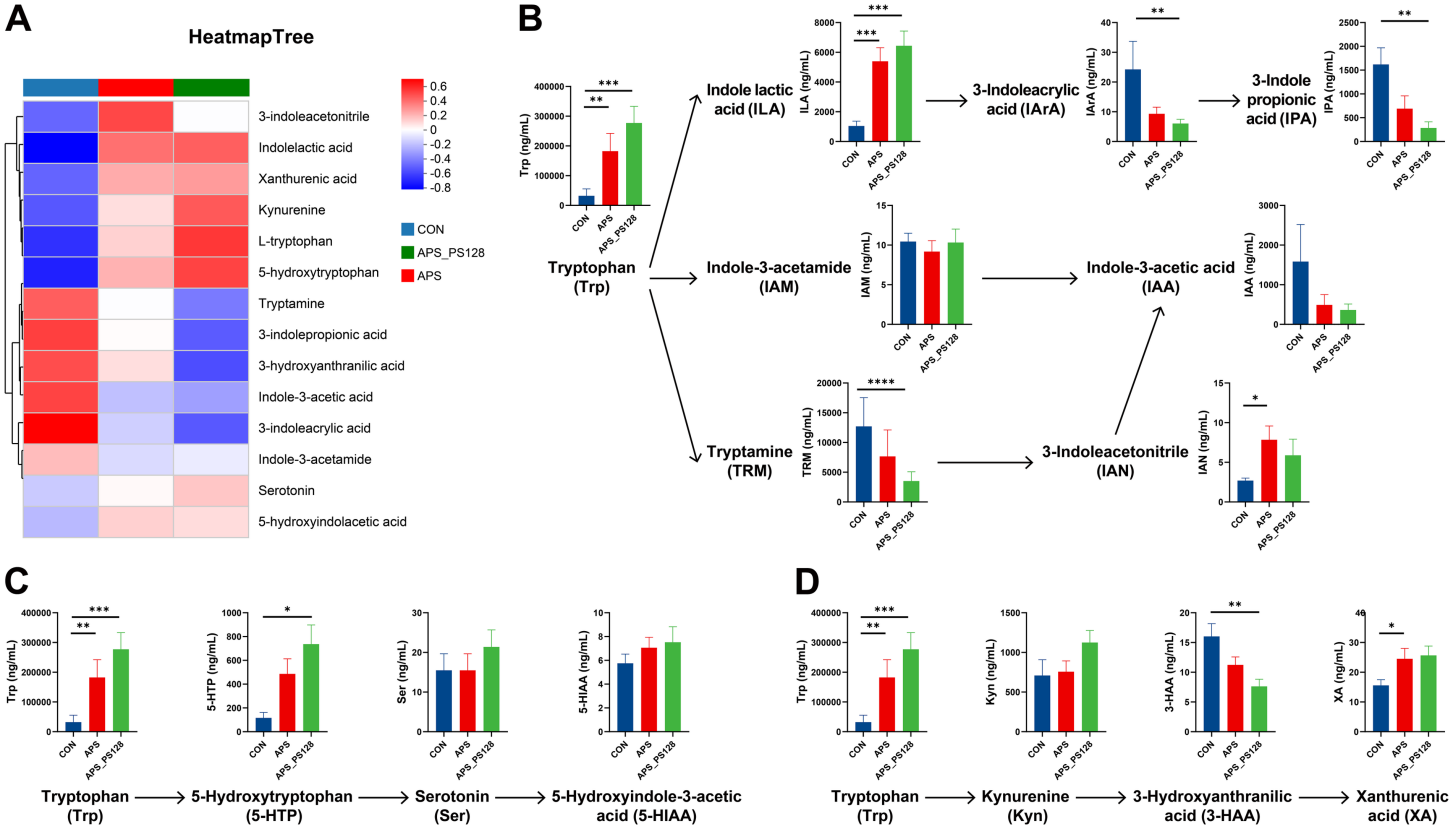
Effects of APS, and APS_PS128 on tryptophan (Trp) related metabolites. **(A)** Heatmap showing the average concentration of 14 Trp related metabolites; **(B)** indole pathway; **(C)** serotonin pathway; **(D)** kynurenine pathway. The color from red to blue represents the change in average concentration from high to low. Data are presented as mean ± SEM, and differences are denoted as follows: *0.01 < *p* ≤ 0.05, **0.001 < *p* ≤ 0.01, ***0.0001 < *p* ≤ 0.001, and ****p ≤ 0.0001.

Figure 3A showed a heatmap of the average concentrations of 14 Trp related metabolites. In the indole pathway (Figure 3B), the levels of Trp in the APS group (*p* < 0.01) and the APS_PS128 group (*p* < 0.001) was significantly higher than the CON group. Similar trends were observed in indole lactic acid (ILA), with the APS group (*p* < 0.001) and the APS_PS128 group (*p* < 0.001) showing significantly higher levels than the CON group. The levels of 3-Indoleacetonitrile (IAN) in the APS group was significantly higher than that in the CON group (*p* < 0.05). In contrast, the levels of 3-Indoleacrylic acid (IArA), 3-Indole propionic acid (IPA), and tryptamine (TRM) in the APS_PS128 group were significantly lower than the CON group (all *p* < 0.01). In the serotonin pathway (Figure 3C), the levels of 5-hydroxytryptophan (5-HTP) in the APS_PS128 group (*p* < 0.05) was significantly higher than the CON group. In the kynurenine pathway (Figure 3D), the levels of 3-Hydroxyanthranilic acid (3-HAA) in the APS_PS128 group (*p* < 0.01) was significantly lower than the CON group, while the levels of Xanthurenic acid (XA) in the APS group (*p* < 0.05) was significantly higher than the CON group. Notably, there was no significant difference in Trp related metabolites between the APS_PS128 group and the APS group (*p* > 0.05).

### 3.4 Correlations between gut microbiota and differential metabolites

**Figure 4:**
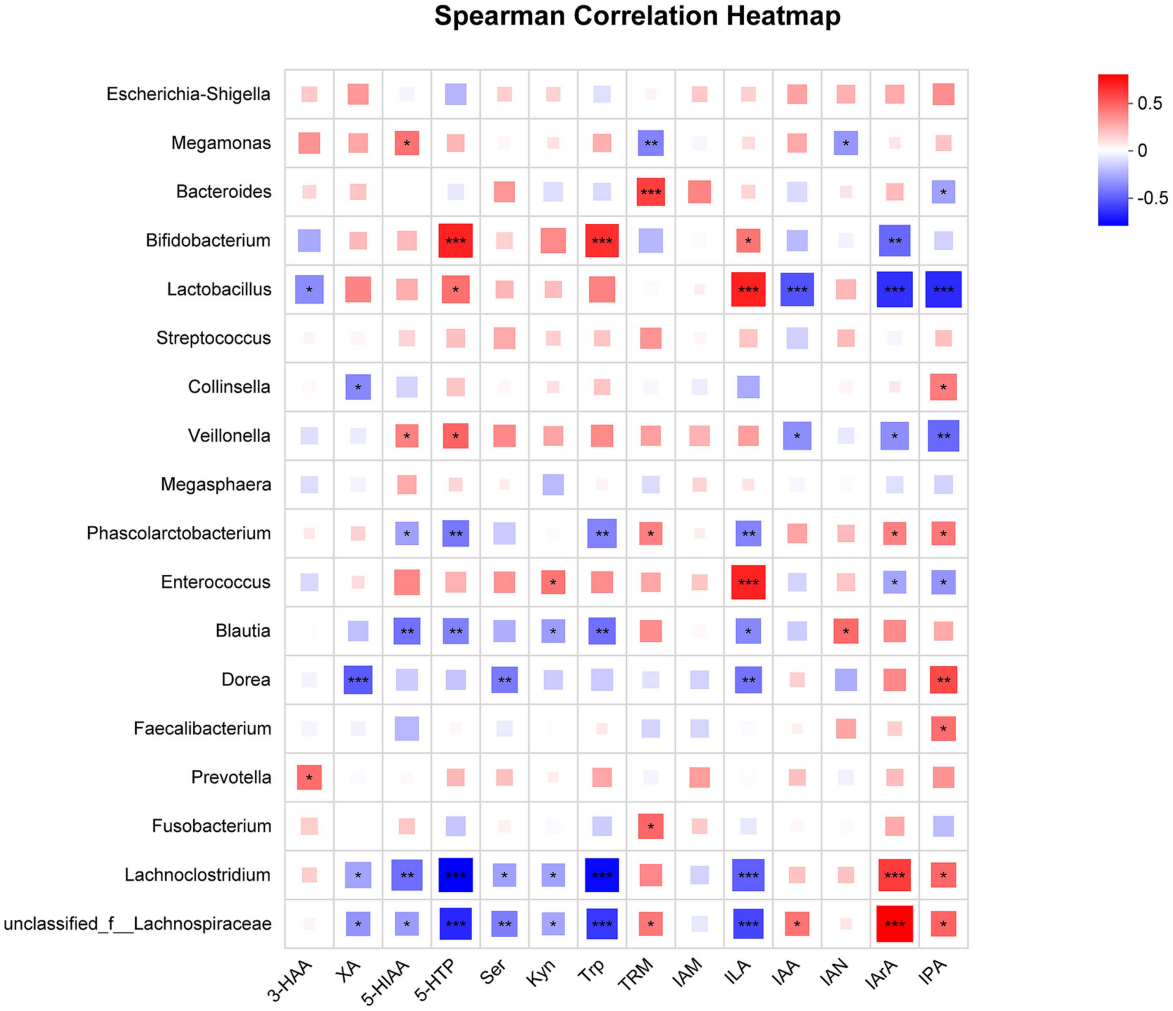
Spearman correlation heatmap between differential metabolites and microbiota at the genus level. The color from red to blue represents the changes of the correlation coefficient r from greater to lower. Differences are denoted as follows: *0.01 < *p* ≤ 0.05, **0.001 < *p* ≤ 0.01, ***0.0001 < *p* ≤ 0.001, and **** *p* ≤ 0.0001.

Figure 4 shows spearman correlation analysis results between differential metabolites and gut microbiota at the genus level. *Bifidobacterium* showed a positive correlation with ILA (*r* = 0.327, *p <* 0.05), Trp (*r* = 0.523, *p <* 0.001), and 5-HTP (*r* = 0.556, *p <* 0.001), whereas negatively correlated with IArA (*r* = −0.460, *p <* 0.01). *Lactobacillus* was positively correlated with 5-HTP (*r* = 0.342, *p <* 0.05) and ILA (*r* = 0.559, *p <* 0.001), while negatively correlated with 3-HAA (*r* = −0.364, *p <* 0.05), IAA (*r* = −0.519, *p* ≤ 0.001), IArA (*r* = −0.612, *p <* 0.001) and IPA (*r* = −0.632, *p <* 0.001). *Lachnoclostridium* was positively correlated with IPA (*r* = 0.366, *p <* 0.05) and IArA (*r* = 0.483, *p <* 0.001), whereas negatively correlated with XA (*r* = −0.300, *p <* 0.05), Kyn (*r* = −0.306, *p <* 0.05), Ser (*r* = −0.309, *p <* 0.05), 5-HIAA (*r* = −0.445, *p <* 0.01), ILA (*r* = −0.483, *p <* 0.001), 5-HTP (*r* = −0.746, *p <* 0.001), and Trp (*r* = −0.724, *p <* 0.001).

## 4 Discussion

Our study examined the effects of APS and APS_PS128 on gut microbiota and Trp related metabolites of MDD patients using *in vitro* fermentation model. After 48 h of *in vitro* fermentation, APS fermentation increased the abundance of *Bifidobacterium* and decreased the abundance of *Lachnoclostridium*. APS also increased total gas production and levels of ILA, Trp, and 5-HTP. Compared with APS, APS_PS128 increased the abundance of *Lactobacillus*, reduced total gas production and percentages of CO_2_, H_2_, and H_2_S, and to some extent increased the abundance of the levels of ILA, Trp, and 5-HTP, although not statistically significant. These results indicate that APS fermentation could regulate gut microbiota structure and Trp related metabolites of MDD patients; Compared to APS, APS and PS128 synbiotic fermentation could reduce gas production but shows limited ability to modulate gut microbiota structure or Trp related metabolite in MDD patients. Correlation analysis further showed *Bifidobacterium* was positively correlated with ILA, Trp, and 5-HTP; On the contrary, *Lachnoclostridium* was negatively correlated with ILA, Trp, and 5-HTP. We speculate that APS might increase ILA, Trp, and 5-HTP levels by promoting the abundance of *Bifidobacterium* and inhibiting the abundanc of *Lachnoclostridium* in MDD patients. This study provides evidence for the application of APS as potential therapeutic strategies for MDD.

Gas production is one of the main side effects of consuming prebiotics and probiotics (31) and excessive gas production can lead to flatulence issues (32). Excessive CO_2_ output is considered detrimental to human health; it can directly alter the volume of the intestinal microenvironment and indirectly promote intestinal peristalsis by reducing intestinal pH (33). The enrichment of H_2_S also has adverse effects on health. Low H_2_S production has a protective effect on intestinal epithelial cell regeneration, including anti-inflammatory and cell regeneration (34), while high H_2_S production is detrimental to intestinal epithelial cell, including cell apoptosis and villus contraction (35). Our study found that APS fermentation increased total gas production, while APS_PS128 fermentation reduced total gas production, and percentages of CO_2_, H_2_, and H_2_S compared to APS fermentation. This indicates that APS with PS128 synbiotics fermentation could reduce gas production during APS fermentation alone.

Previous studies have found depression is closely related to intestinal dysbiosis. High enrichment of conditionally pathogenic bacteria such as *Lachnoclostridium*, *Eggerthella*, *Enterococcus,* and *Flavonifractor* can induce the development and progression of MDD (36). Conversely, a decrease level of commensal or beneficial bacteria such as *Lactobacillus*, *Bifidobacterium*, and *Butyricicoccus* has been reported in MDD patients (36, 37). Supplementing with *Lacticaseibacillus paracasei strain* Shirota for 12 weeks significantly improved the mood of MDD patients, and the improvement of depressive symptoms was related to the gut microbiota such as *Bifidobacterium* (38). Studies have also shown that supplementing with *Lacticaseibacillus plantarum* PS128 is effective for patients with anxiety (39), depression (23), and insomnia (24). In our study, we found that APS fermentation increased the abundance of *Bifidobacterium* and decreased the abundance of *Lachnoclostridium* in the fecal microbiota of MDD patients. Compared with APS, APS_PS128 fermentation only increased the abundance of *Lactobacillus*. Based on previous reports and our study findings, we found APS fermentation increased conditionally pathogenic bacteria and reduce commercial or beneficial bacteria in MDD patients, with great potential for the treatment of MDD. However, PS128 appears to have limited ability to utilize APS in MDD patients.

A growing number of evidence indicates that gut microbiota regulates tryptophan metabolism to affect host’s mental health (40, 41). Tryptophan (Trp) is mainly obtained from food, and its main metabolic pathways include indole pathway, serotonin pathway, and kynurenine pathway. Previous studies have found that tryptophan and its metabolites are closely related to depression. A meta-analysis showed that plasma Trp levels were reduced in MDD patients compared to healthy controls (42). The conversion of Trp into indole derivatives such as ILA, which exert biological effects on the host. A clinical experiment showed serum ILA levels in MDD patients were significantly reduced compared to healthy controls (43), and an animal experiment showed that an increase in ILA levels was associated with the improvement of depressive symptoms (8). 5-HTP is converted from Trp and subsequently converted into Ser. Animal experiments showed that *Lacticaseibacillus rhamnosus* KY16 improved depressive like behavior by promoting intestinal secretion of 5-HTP (44), and the administration of 5-HTP restored gut microbiota dysbiosis in depressed mice (45). A meta-analysis found a significant positive effect of 5-HTP supplementation in the treatment of depression (46). To sum up, these studies found depressed patients/mice may have lower levels of ILA, Trp and 5-HTP, and supplementing with these substances may improve depressive symptoms. This is consistent with our study findings that APS fermentation increased the production of ILA, Trp, and 5-HTP, indicating APS is a potential strategy for treating depression. Compared with APS, APS_PS128 fermentation increased the abundance of *Lactobacillus* (*p* < 0.05), and to some extent increased the levels of ILA, Trp, and 5-HTP, although not statistically significant (*p* > 0.05). The above results suggested that APS fermentation could alter the Trp related metabolites of MDD patients, while the consumption of PS128 seemed to have limited ability to utilize APS in MDD patients. We speculate that this may be because APS is a complex water-soluble heteropolysaccharide, and PS128 may lack the ability to break down complex sugar chains in APS, thereby limiting its utilization of APS.

Correlation analysis further confirmed the correlations between intestinal microbiota and differential metabolites in MDD patients. Interestingly, correlation analysis showed *Lachnoclostridium* was negatively correlated with ILA, Trp, and 5-HTP; *Bifidobacterium* was positively correlated with ILA, Trp, and 5-HTP. This is consistent with previous reports that bifidobacteria supplementation will increase the levels of ILA in the serum and feces of both humans and depressed mice (8). Therefore, we infer that the APS might upregulate ILA, Trp, and 5-HTP levels by increasing the abundance of *Bifidobacterium* and reducing the abundance of *Lachnoclostridium* in MDD patients.

To our knowledge, this is the first study to use an *in vitro* simulated fermentation model to elucidate the effects of APS on gut microbiota and Trp related metabolites of MDD patients. This is also the first study to explore whether psychobiotic (PS128) can improve the utilization of prebiotics (APS) in MDD patients. Notably, our study subjects were first-episode and drug naïve MDD patients, which excluded the influence of confounding factors such as medication and frequency of episodes. However, this study had some limitations. First, *in vitro* experiments may not completely yield to *in vivo* conditions and there may be differences between the results of *in vitro* research based on simulated fermentation and those based on the actual environment in the human gut (47). Second, we did not include healthy individuals, so we cannot conclude that APS can reverse changes in gut microbiota and metabolites in patients with depression. Third, changes in pH value during fermentation could influence microbial activity and results, but we did not monitor the pH value during incubation. Finally, our study has a small sample size and an imbalanced gender distribution, which impairs the ability to generalize results and may not be applicable to both genders. Despite the inherent limitations, our findings provide certain value for the research on the application of prebiotics and probiotics in the treatment of depression. Future studies are necessary to conduct animal and clinical experiments to validate the results of APS in this study. It is also necessary to explore the optimal combination of prebiotics and probiotics for treating MDD.

## 5 Conclusion

Our study showed APS increased the abundance of *Bifidobacterium* and decreased the abundance of *Lachnoclostridium* in the fecal microbiota of MDD patients after 48 h of *in vitro* fermentation. APS also increased total gas production and levels of ILA, Trp and 5-HTP. Compared with APS, APS with PS128 synbiotics increased the abundance of *Lactobacillus*, reduced total gas production and percentages of CO_2_, H_2_, and H_2_S, and to some extent increased the levels of ILA, Trp, and 5-HTP, although not statistically significant. Correlation analysis further showed *Bifidobacterium* was positively correlated with ILA, Trp, and 5-HTP; On the contrary, *Lachnoclostridium* was negatively correlated with ILA, Trp, and 5-HTP. We speculate that APS might increase ILA, Trp, and 5-HTP levels by promoting the abundance of *Bifidobacterium* and inhibiting the abundanc of *Lachnoclostridium* in MDD patients. All these results suggest that APS could regulate gut microbiota structure and Trp related metabolites in MDD patients; Compared to APS, APS and PS128 synbiotic fermentation could reduce gas production but shows limited ability to modulate gut microbiota structure or Trp related metabolite in MDD patients. Future animal and clinical experiments are needed to validate the results of APS in this study, and further explore the optimal combination of prebiotics and probiotics for treating depression.

## Data Availability

The datasets presented in this study can be found in online repositories. The names of the repository and accession number can be found in the article.

## Footnotes

^1^ https://cloud.majorbio.com
